# Histone demethylase GASC1 - a potential prognostic and predictive marker in invasive breast cancer

**DOI:** 10.1186/1471-2407-12-516

**Published:** 2012-11-14

**Authors:** Bozena Berdel, Kaisa Nieminen, Ylermi Soini, Maria Tengström, Marjo Malinen, Veli-Matti Kosma, Jorma J Palvimo, Arto Mannermaa

**Affiliations:** 1 Department of Pathology and Forensic Medicine, Institute of Clinical Medicine, University of Eastern Finland; Cancer Center of Eastern Finland, P.O. Box 1627, FI-70211, Kuopio, Finland; 2 Department of Clinical Pathology, Imaging Center, Kuopio University Hospital, P.O. Box 1777, FI-70211, Kuopio, Finland; 3 Biocenter Kuopio, University of Eastern Finland, P.O. Box 1627, FI-70211, Kuopio, Finland; 4 Cancer Center, Kuopio University Hospital, P.O. Box 1777, FI-70211, Kuopio, Finland; 5 Institute of Biomedicine/Medical Biochemistry, University of Eastern Finland, Kuopio, Finland

**Keywords:** Epigenetics, GASC1, Breast cancer, Survival, Tissue microarrays

## Abstract

**Background:**

The histone demethylase GASC1 (JMJD2C) is an epigenetic factor suspected of involvement in development of different cancers, including breast cancer. It is thought to be overexpressed in the more aggressive breast cancer types based on mRNA expression studies on cell lines and meta analysis of human breast cancer sets. This study aimed to evaluate the prognostic and predictive value of GASC1 for women with invasive breast cancer.

**Methods:**

All the 355 cases were selected from a cohort enrolled in the Kuopio Breast Cancer Project between April 1990 and December 1995. The expression of GASC1 was studied by immunohistochemistry (IHC) on tissue microarrays. Additionally relative *GASC1* mRNA expression was measured from available 57 cases.

**Results:**

In our material, 56% of the cases were GASC1 negative and 44% positive in IHC staining. Women with GASC1 negative tumors had two years shorter breast cancer specific survival and time to relapse than the women with GASC1 positive tumors (p=0.017 and p=0.034 respectively). The majority of GASC1 negative tumors were ductal cases (72%) of higher histological grade (84% of grade II and III altogether). When we evaluated estrogen receptor negative and progesterone receptor negative cases separately, there was 2 times more GASC1 negative than GASC1 positive tumors in each group (chi2, p= 0.033 and 0.001 respectively). In the HER2 positive cases, there was 3 times more GASC1 negative cases than GASC1 positives (chi2, p= 0.029). Patients treated with radiotherapy (n=206) and hormonal treatment (n=62) had better breast cancer specific survival, when they were GASC1 positive (Cox regression: HR=0.49, p=0.007 and HR=0.33, p=0.015, respectively). The expression of *GASC1* mRNA was in agreement with the protein analysis.

**Conclusions:**

This study indicates that the GASC1 is both a prognostic and a predictive factor for women with invasive breast cancer. GASC1 negativity is associated with tumors of more aggressive histopathological types (ductal type, grade II and III, ER negative, PR negative). Patients with GASC1 positive tumors have better breast cancer specific survival and respond better to radiotherapy and hormonal treatment.

## Background

Breast cancer is a heterogeneous disease with different histopathological, molecular and clinical characteristics. Moreover, there is a wide variation in the progress of breast cancer in patients of the same age and with tumors of comparable clinical extent. The discovery of molecular markers, such as estrogen, progesterone and HER2 receptors has facilitated the classification of tumors and led to discovery of new cancer therapies. However, the selection of patients for appropriate adjuvant therapies still encounters difficulties [1]. Therefore there is an urgent need for novel diagnostic, prognostic and predictive markers which would make easier selection of patients for adjuvant therapies and possibly open novel perspectives for more efficient therapeutic strategies.

Nowadays epigenetics is making an increasingly important impact in cancer research [2-4]. Epigenetic research has not only provided novel insights into the molecular mechanisms of cancer, but it has also revealed useful diagnostic, prognostic and predictive biomarkers [5-7]. In addition to epigenetic modifications like DNA methylation and nucleosome positioning, histone modification patterns are altered in human tumors. However, methodological difficulties prevent use of altered histone modification profiles found in cancer as biomarkers [8]. Therefore, there is a considerable interest in understanding histone modifier genes and their products. One of these genes is GASC1 (gene amplified in squamous cell carcinoma 1; aliases: JMJD2C, JHDM3C, KDM4C) which codes a histone demethylase for di- and trimethylated lysine 9 and 36 on histone H3 (H3K9me3/2 and H3K36me3/2) [9,10]. H3K9me3/2 mark is generally associated with transcriptional repression and the formation of heterochromatin, while H3K36me3/2 is associated with transcriptionaly active genes and it is believed to play an important role in the suppression of incorrect transcription [10,11]. Because of its dual role in modifying H3 either by removing the repressive H3K9me3/2 or the active H3K36me3/2 factor, GASC1 has been considered to be a fine-tuning regulator of gene expression in normal development and differentiation as well as in cancer development and progression [12-14]. The involvement of *GASC1* in development of breast cancer has been well documented in cell lines by Liu et al. [13] and Wu at al. [15]. They demonstrated that *GASC1* is amplified and overexpressed in multiple breast cancer cell lines, it causes transformation of immortalized, non-transformed mammary epithelial cells, regulates expression of genes responsible for stem cells self-renewal and may be linked to the stem cell phenotypes in breast cancer. Additionally, they have found that the *GASC1* is overexpressed in aggressive, basal-like breast cancers compared with non basal-like breast cancers.

Moreover, an oncogenic role of GASC1 has been documented in prostate cancer where it enhances the transcription of androgen receptor-dependent genes and cell proliferation by interaction with ligand-bound androgen receptor [16]. GASC1 also plays an important role in normal development and differentiation by regulating expression of pluripotency genes, including NOTCH1, NANOG, Sox2 and Pou5 [17,18].

As far as we are aware, this marker has not been evaluated by immunohistochemistry in human breast tumors. Consequently, this study aimed at determining the relevance of GASC1 demethylase in the prognosis of invasive breast cancer progression and in the prediction of responses to particular adjuvant treatments in material from a large cohort of patients with a detailed clinical and histopathological classification of tumors and up to 20 years of follow-up [19]. Additionally, we investigated whether this marker could be utilized for more detailed classification of invasive breast cancer.

## Methods

Our initial material consisted of 392 breast cancer cases selected from a cohort enrolled into the Kuopio Breast Cancer Project in the Kuopio University Hospital, Kuopio, Finland between April 1990 and December 1995 [20,21].

**Figure 1:**
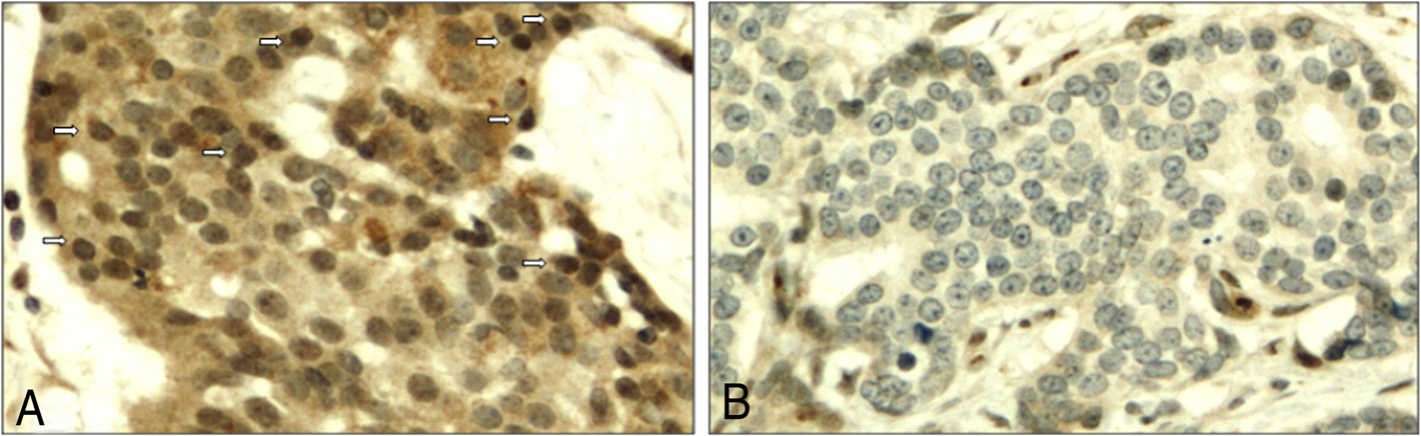
**Expression of GASC1 in invasive breast carcinoma of ductal type. **(**A**) Positive immunostaining in nuclei of epithelial cells (arrows; immunoscores: 3 for the nuclear number and 3 for intensity of nuclear staining), positive staining visible in cytoplasm was not taken into account. Original magnification of x200. (**B**) Negative GASC1 immunostaining in nuclei of epithelial carcinoma cells. Original magnification of x200.

The tumor samples from these patients were fixed in 10% buffered formalin and embedded in paraffin. The histological diagnosis was confirmed by reviewing one to four original sections of the primary tumor. From the total material (392 tumors), we excluded 37 benign and in situ cases. In the remaining 355 cases of invasive breast tumors without distant metastases, we evaluated GASC1 expression by immunohistochemical (IHC) staining in the nuclei of the tumor epithelial cells (Figure 1). Further we analyzed how the GASC1 status would influence the breast cancer specific survival and the time to relapse.

In the analyzed by IHC material, 198 (55.8%) cases were GASC1 negative and 157 (44.2%) GASC1 positive. In this cohort, 105 patients had died of breast cancer, 106 patients had died from other causes than breast cancer and 144 patients were still alive at the time of analysis. The mean follow up time at the cut-off point in February 2011 was 10.6 years, ranging from 0.1 to 20.4 years. Sixty eight patients had undergone resection, 285 were treated with mastectomy, and two patients did not undergo surgery. Postoperative radiotherapy was given to 206 patients. Altogether 62 patients had received adjuvant tamoxifen, and 69 patients were treated with adjuvant chemotherapy, mainly the intravenous CMF regimen (500 mg/m^2^, methotrexate 40 mg/m^2^, 5-fluorouracil 500 mg/m).

The tissue microarray (TMA) was constructed as described previously [22]. The diagnosis of the cases was based on the World Health Organization (WHO), classification of breast and female genital organs [23]. The presence of metastases was determined at the time of the operation. The collection of the material and the clinical features of the patients have been described in a previous study [21]. The research was approved by the ethical committee of University of Kuopio/University of Eastern Finland and Kuopio University Hospital.

### Immunohistochemistry for GASC1

Immunohistochemical staining was performed on 4 μm-thick sections cut, from TMA block. After deparaffinization and rehydration, the sections were heated in a microwave oven for 3 × 5 min in citrate buffer (pH 6.0). Then they were treated for 5 min with 5% hydrogen peroxide to block endogenous peroxidase. Next, the sections were incubated for 35 min at room temperature in 1.5% normal serum diluted in PBS to block non-specific binding. After that, the sections were incubated overnight at 4°C with the mouse monoclonal anti – GASC1 antibody (Origene, TA 500587) at dilution 1:100. The slides were then incubated with a biotinylated secondary antibody (35 min) and avidin-biotin-peroxidase complex (45 min) (ABC Vectastain Mouse Elite Kit, Vector Laboratories, Burlingame, CA, USA). After each step of the immunostaining procedure the slides were rinsed with PBS. The color was developed with diaminobenzidine tetrahydrochloride (DAB) (Sigma, St. Louis, MO, USA). The slides were counterstained with Mayer’s haematoxylin, washed, dehydrated, cleared and mounted with Depex (BDH, Poole, UK). In the negative controls, the primary antibody was omitted.

The immunoreactivity for GASC1 was analyzed in the nuclei of epithelial tumor cells taking into account the number of positively stained nuclei and intensity of staining. The number of positively stained nuclei was semiquantified as follows:

0-5% of nuclei stained = (0)

5-25% of nuclei stained = (1)

25-75% of nuclei stained = (2)

75-100% of nuclei stained = (3)

The intensity of nuclear staining was evaluated on a four grade scale:

0 - negative

1 - weak

2 - moderate

3 - intensive

The evaluation was performed on two separate array cores (A and B) by two pathologists (YS, BB) blinded to outcome (Figure 1). The results in series A and B were similar (substantial inter-series agreement was achieved; kappa for both nuclear number and intensity was 0.7; p=0.000). The final score was obtained by combining these results into four groups. Tumors were designated as negative if their scores from both series A and B were 0. Tumors were designated as positive if their score from at least one series was positive.

### mRNA expression analysis

For this study from this cohort there were 57 samples available with invasive breast tumors. RNA was isolated from fresh frozen tissue stored at −70°C using Protein and RNA Isolation System for Small RNAs (mirVana^TM^ Paris^TM^). We used High Capacity cDNA Reverse Transcription Kit to synthesize cDNA (Applied Biosystems, Foster City, USA) following the manufacturer’s instructions. The endogenous control gene was chosen by the investigation conducted by McNeill et al. [24] where they determined PPIA to be the best choice for breast cancer mRNA analysis. Next we performed quantitative real-time RT-PCR using gene specific TaqMan® Gene Expression Assays (Applied Biosystems). Relative gene expression values were calculated as the ratio between the target gene and the endogenous control PPIA, obtained for each sample from the standard curves. Finally, the values greater than the mean were designated as positives and lower than the mean as negatives.

### Statistical analysis

The expression of GASC1 in different groups was compared using chi-squared test. In breast cancer specific survival analysis, the end point was death from breast cancer with deaths from other causes being censored, whereas in time to relapse analysis, the end point was breast cancer recurrence, either local or distant. Overall survival was calculated from the date of diagnosis to the date of death or the last follow-up date. Kaplan-Meier analysis was applied to estimate breast cancer specific survival and overall survival according to the adjuvant treatment; different groups were compared with the log-rank test. Multivariate analyses were conducted with the Cox regression model. The statistical analyses were performed using SPSS version 17.0 (SPSS Inc., Chicago, IL, USA). *p* < 0.05 was considered statistically significant.

## Results

### GASC1 IHC negativity is an independent prognostic factor of poorer breast cancer specific survival

**Figure 2:**
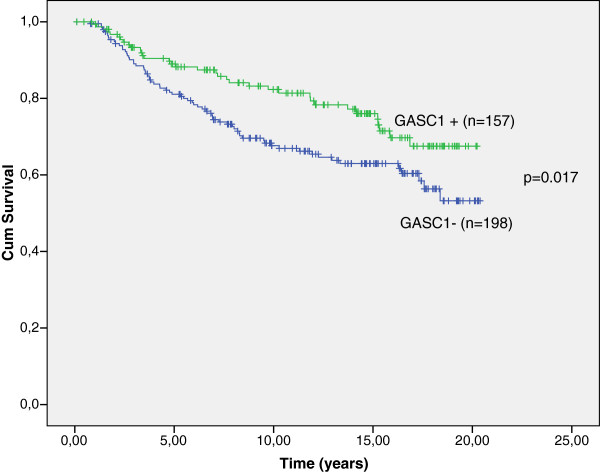
**Breast cancer specific survival by Kaplan-Meier analysis. **Overall, the patients with GASC1 immunopositive tumors have better survival than the patients with GASC1 negative tumors (p=0.017 Log Rank; p=0.012, Breslow; p=0.013, Tarone-Ware).

Overall, women with GASC1 negative tumors (n=198) had two years shorter breast cancer specific survival than the women with GASC1 positive tumors (n=157; Table 1, Figure 2). Stratification according to clinical parameters revealed that the GASC1 negative women, over 55 years of age, with tumor size T2, T3 or T4, with positive nodal status and with clinical stage II, III or IV had a significantly poorer survival than the GASC1 positive ones. In patients aged 55 years or younger and patients with clinical stage I, the GASC1 status did not influence the survival time (Table 1).

**Table 1:** Breast cancer specific survival by Kaplan-Meier analysis according to clinical parameters

**Variable**	**n**	**Means of survival time (years) [n]**		**p-value**
		**GASC1 negative**	**GASC1 positive**	
Overall	355	14.6 [198]	16.6 [157]	**0.017**
Age				
<=55	172	15.0 [107]	16.3 [65]	0.292
>55	183	14.0 [91]	16.4 [92]	**0.017** [0.019]
Tumor size				
T1	168	16.6 [92]	17.0 [76]	0.376
T2+T3+T4	187	12.7 [106]	15.8 [81]	**0.010** [0.749]
Nodal status				
negative	193	17.1 [111]	18.0 [82]	0.109
positive	162	11.4 [87]	14.8 [75]	**0.012** [0.520]
Clinical stage				
I	120	17.8 [66]	17.8 [54]	0.579
II+III+IV	230	12.8 [130]	15.9 [100]	**0.005** [0.820]

The p-values in the first column are calculated using Log Rank (Mantel-Cox) test for equality of survival distributions for the different levels of GASC1 (significant p-values in bold). The p-values in the second column in the squared brackets are calculated using Fisher’s exact test to examine if there is a difference in proportion of GASC1 negative and positive cases in groups of different clinical parameters. n – number of cases.

**Figure 3:**
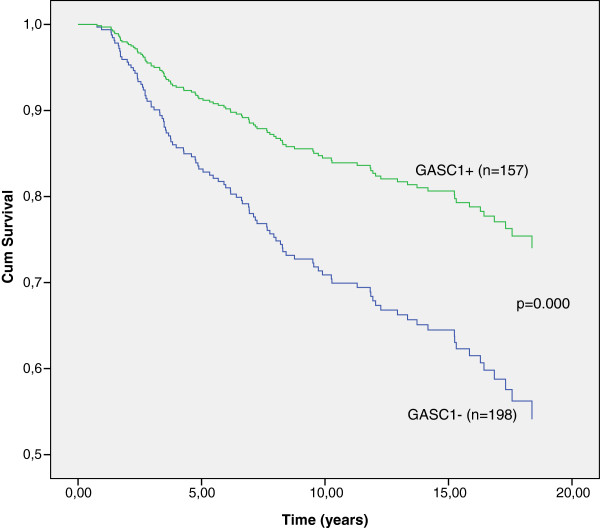
**Breast cancer specific survival analysis by Cox regression. **After adjusting the model to confound for the age at diagnosis, nodal status, size of tumor, and clinical stage, GASC1 positive patients have better survival than GASC1 negative. The p-value for the model is 0.000 and for the GASC1 status 0.001.

After including the above clinical parameters as covariates in the Cox regression analysis, we confirmed that GASC1 negativity was an independent factor predicting poorer breast cancer specific survival, equal to the positive nodal status (p=0.001, Table 2, Figure 3). In this analysis tumor size (T2, T3 and T4) and clinical stage (II, III and IV) had no effect on breast cancer specific survival and time to relapse possibly, because there were only 34 patients (9.6%) with T3 and T4 tumors and 42 patients (12%) at Stage III and Stage IV in our material. Probably, this number of cases was not sufficient to show a significant influence on survival in multivariate analysis. However, in univariate analysis the patients with T1 and at Stage I survived significantly better and had significantly longer time to relapse than the patients with more advanced disease. Similarly, in bivariate analysis, where as the second variable in addition to tumor size or Stage was entered GASC1 status, tumor size or stage and GASC1 status had a significant effect on the breast cancer specific survival and time to relapse.

**Table 2:** Analysis of breast cancer specific survival and time to relapse in the whole group of patients by Cox regression

**Variable**	**n**	**Breast cancer specific survival**		**Time to relapse**	
		**HR (95% CI)**	**p-value**	**HR (95%CI)**	**p-value**
Age					
<=55	172				
>55	183	0.948 (0.638-1.408)	0.791	0.993 (0.698-1.413)	0.969
GASC1 status					
positive	157				
negative	198	2.040 (1.345-3.092)	**0.001**	1.766 (1.230-2.535)	**0.002**
Nodal status					
negative	193				
positive	162	3.177 (1.768-5.710)	**0.000**	2.835 (1.702-4.723)	**0.000**
Tumor size					
T1	168				
T2+T3+T4	187	0.621 (0.356-1.080)	0.092	0.707 (0.431-1.161)	0.171
Clinical stage					
I	120				
II+III+IV	230	1.107 (0.456-2.687)	0.823	1.092 (0.511-2.335)	0.820
unknown	5				

n – number of cases, HR – hazard ratio.

### GASC1 IHC negative cases have a shorter time to relapse than the GASC1 positive cases

**Figure 4:**
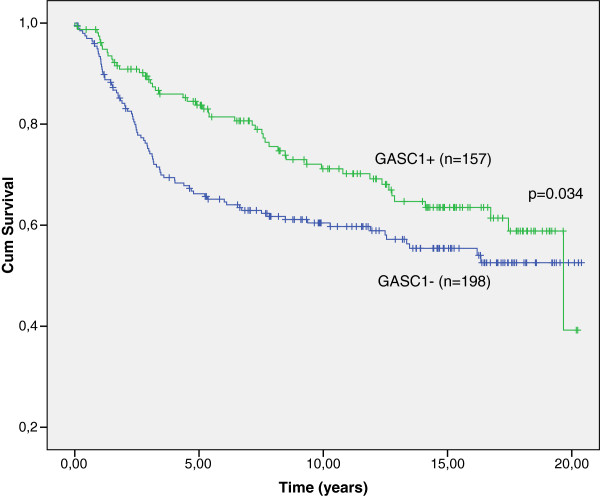
**Time to relapse by Kaplan-Meier analysis. **Overall, the patients with GASC1 immunopositive tumors have a longer time to relapse than the patients with GASC1 negative tumors (p=0.034, Log Rank; p=0.005, Breslow; p=0.010 Tarone-Ware).

**Figure 5:**
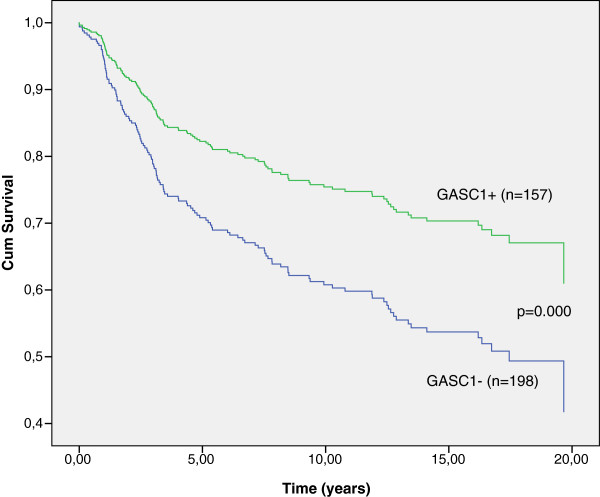
**Time to relapse analysis by Cox regression. **GASC1 negative cases have a shorter time to relapse than GASC1 positive cases. The following covariates were included into the model: age at diagnosis, nodal status, size of tumor, clinical stage and GASC1 status. The p-value for the model is 0.000 and for the GASC1 status 0.002.

Overall, there were 132 cases with relapse and breast cancer was the cause of death of 105 women. Eleven women with a relapse died from other causes and 16 were still alive at the time of analysis. Women with GASC1 negative tumors had a shorter time to relapse than the women with GASC1 positive tumors (Table 3, Figure 4). After stratification according to the clinical parameters, the results were similar to those obtained in breast cancer specific survival analysis, which suggests that among subjects with more advanced stages of the disease, GASC1 negative cases had poorer prognosis (Table 3). Cox regression analysis verified that the GASC1 negativity is an independent factor predicting a shorter time to relapse in women with invasive breast cancer (p=0.002, Table 2, Figure 5).

**Table 3:** Time to relapse by Kaplan-Meier analysis

**Variable**	**n**	**Means of survival time (years) / n**		**p-value, Log Rank**
		**GASC1 negative**	**GASC1 positive**	
Overall	355	12.9/198	14.8/157	**0.034** (0.005B, 0.010T-W)
Age				
<=55	172	12.9/107	14.9/65	0.168
>55	183	12.9/ 91	14.5/92	0.098, (0.035 B, 0.052 T-W)
Tumor size				
T1	168	14.8/ 92	15.6/76	0.331
T2+T3+T4	187	11.1/106	13.9/81	**0.040** (0.013B, 0.018 T-W)
Nodal status				
negative	193	15.5/111	16.6/82	0.135
positive	162	9.1/ 87	12.8/75	**0.013** (0.002B, 0.004 T-W)
Clinical stage				
I	120	16.2/ 66	16.5/ 54	0.580
II+III+IV	230	11.1/130	14.0/100	**0.014** (0.003 B, 0.005 T-W)

p-value for test of equality of survival distributions for the different levels of GASC1, B: Breslow (Generalized Wilcoxon),T-W: Tarone –Ware, n – number of cases.

### GASC1 IHC negative cases are more likely to have a relapse of breast cancer and to suffer from more aggressive tumors than the GASC1 positive cases

Among patients with a relapse (n=132) there were 26% more GASC1 negative cases (n=83) than GASC1 positive (n=49), while among patients without relapse (n=223) the difference between GASC1 negative (n=115) and positive (n=108) cases was only 4% (chi2, p=0.038).

The majority of GASC1 negative tumors were ductal (72 %) of higher histological grade (84% of grade II and III altogether). The GASC1 negative tumors significantly differ in terms of histological type and grade, estrogen receptor (ER), progesterone receptor (PR) and HER2 status from GASC1 positive tumors (p=0.005, p=0.000, p=0.033, p=0.001 and p=0.029 respectively; Table 4). The aforementioned results pointed to the possibility that GASC1 negativity might only be a marker for aggressive tumor subtype but not an independent marker of breast cancer specific survival. To check this possibility, we performed the Kaplan-Meier survival analysis in the above groups of patients, which showed that the GASC1 negativity significantly worsened the survival in ductal cases (Log Rank p=0.006), ER positive cases (Log Rank p=0.043) and HER2 negative cases (Log Rank p=0.018; Table 5). Even after adjusting for histological type, histological grade, ER, PR, HER2 and nodal status, GASC1 negativity was significantly associated with poorer breast cancer specific survival (HR=2.0, p=0.004).

**Table 4:** GASC1 immunostaining according to histopathological and molecular parameters

**Variable**	**n**	**GASC1 negative n (%)**	**GASC1 positive n (%)**	**p-value, chi2**
Number of patients	355	198 (55.8)	157 (44.2)	
Histological type				
Ductal	231	143 (62)	88 (38)	**0.005**
Lobular	66	31 (47)	35 (53)	
Rare	58	24 (41)	34 (59)	
Histological grade				
I	84	31 (37)	53 (63)	**0.000**
II	166	84 (51)	82 (49)	
III	103	81 (79)	22 (21)	
unknown	2			
ER status				
negative	77	51 (66)	26 (34)	**0.033**
positive	272	143 (53)	129 (47)	
unknown	6			
PR status				
negative	137	91 (66)	46 (34)	**0.001**
positive	212	103 (49)	109 (51)	
unknown	6			
HER2 status				
negative	290	156 (54)	134 (46)	**0.029**
positive	45	32 (71)	13 (29)	
unknown	20			

n – number of cases.

**Table 5:** Breast cancer specific survival by Kaplan Meier analysis according to histopathological and molecular parameters

**Variable**	**n**	**Means of survival time (years) / n**		**p-value, Log Rank**
		**GASC1 negative**	**GASC1 positive**	
Overall	355	14.6/198	16.6/157	**0.017** (0.012B, 0.013T-W)
Histological type				
Ductal	231	14.5/143	17.3/88	**0.006**
Others	124	14.8/ 55	15.4/69	0.608
Tumor grade				
I	84	16.5/ 31	17.0/ 53	0.415
II+III	269	14.3/165	16.0/104	0.087
unknown	2			
ER status				
negative	77	13.4/ 51	15.2/ 26	0.322
positive	272	15.0/143	16.7/129	**0.043** (0.027 B)
unknown	6			
PR status				
negative	137	13.6/ 91	16.5/ 46	0.061 (0.042 B)
positive	212	15.4/103	16.5/109	0.251
unknown	6			
HER2 status				
negative	290	15.3/156	17.4/134	**0.018** (0.003 B)
positive	45	12.1/ 32	13.0/ 13	0.704
unknown	20			
HER2^-^/ductal/grade II	90	14.4/49	18.1/41	**0.015**
Triple negative	39	14.6/29	18.0/10	0.196

p- value for test of equality of survival distributions for the different levels of GASC1, B: Breslow (Generalized Wilcoxon),T-W: Tarone –Ware, n – number of cases.

### GASC1 IHC positivity is an independent marker for better prognosis in patients treated with radiotherapy or tamoxifen

We also analyzed the predictive value of GASC1 staining according to the adjuvant treatments that the patients were given. There were 206 patients who were treated with adjuvant radiotherapy. Kaplan-Meier analysis detected a better relapse-free survival (Log Rank 0.017) and breast cancer specific survival (Log Rank 0.021) in the patients with GASC1 positive tumors. In the Cox regression analysis after adjusting for age, stage, chemotherapy, hormonal therapy and hormone receptor status, GASC1 positivity was statistically significantly associated with improved relapse-free and better breast cancer specific survival (Table 6). Overall survival was not affected by the GASC1 staining intensity.

**Table 6:** GASC1 and survival of breast cancer patients according to the type of adjuvant treatment

**Cases**	**n**	**RFS**			**BCSS**			**OS**		
		**HR**	**95% CI**	p*	**HR**	**95% CI**	p*	**HR**	**95% CI**	p*
radiotherapy	206									
negative	119	1§			1§			1§		
positive	87	0.52	0.33 to 0.83	**0.006**	0.49	0.29 to 0.82	**0.007**	0.71	0.47 to 1.06	0.097
tamoxifen	62									
negative	25	1†			1†			1†		
positive	37	0.45	0.21 to 0.99	**0.048**	0.33	0.13 to 0.81	**0.015**	0.66	0.34 to 1.29	0.23
chemotherapy	69									
negative	43	1‡			1‡			1‡		
positive	26	0.64	0.29 to 1.38	0.25	0.70	0.30 to 1.64	0.41	0.81	0.36 to 1.82	0.60

RFS = relapse-free survival; BCSS = breast cancer specific survival; OS = overall survival.

*p was based on Cox proportional analysis.

§Reference value; HRs adjusted for age and stage at diagnosis, chemotherapy, hormonal treatment and hormone receptor status.

†Reference value; HRs adjusted for age and stage at diagnosis and radiotherapy.

‡Reference value; HRs adjusted for age and stage at diagnosis, radiotherapy and hormone receptor status.

Altogether sixty two patients with ER positive tumors were treated with tamoxifen as their only adjuvant medical treatment. Of these patients, 44 received also postoperative radiotherapy. The mean duration of tamoxifen therapy was 36 months (range 3–75). Forty three patients received a daily dosage of 20 mg TAM and 17 patients received a daily dosage of 40 mg TAM. The Cox regression analysis revealed improved relapse-free and breast cancer specific survival in those patients with positive GASC1 staining (Table 6).

GASC1 staining status did not have any effect on the survival of patients given chemotherapy as their only medical adjuvant treatment (n=69). Furthermore, there were no significant differences in overall survival in any of the treatment groups (Table 6).

### *GASC1* mRNA expression is in line with the immunohistochemical data

**Figure 6:**
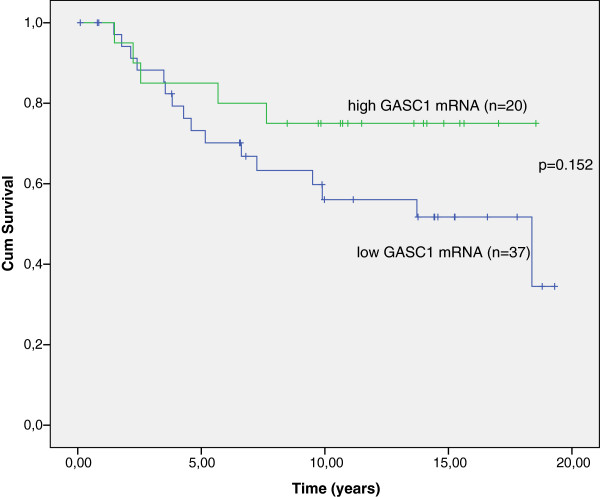
**Breast cancer specific survival by Kaplan-Meier analysis. **Overall, the patients with high expression of *GASC1 *mRNA in tumors have a better survival than patients with low *GASC1 *mRNA expression (p = 0.152, Log Rank).

**Figure 7:**
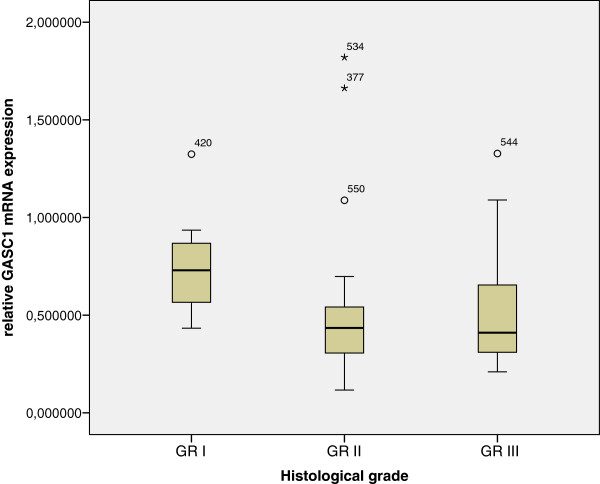
**Graph showing *GASC1 *mRNA expression in tumors of different histological grades. **Boxes represent the 25–75th percentile; whiskers: range; black line: median; black dots: outliers. The highest *GASC1 *mRNA expression is detected in tumors of grade I (GR I; 0.761±0.099). Tumors of grade II (GR II; 0.510±0.070) and III (GR III; 0.510±0.069) show lower *GASC1 *mRNA expression than tumors of grade I. There is no difference in *GASC1 *mRNA expression between tumors of grade II and III. Kruskal-Wallis test, p=0.02. Mann–Whitney test: grade I versus II - p=0.006; grade I versus III - p=0.019, grade II versus III - p=0.821.

The expression of *GASC1* mRNA was evaluated in 57 available cases from the material used in tissue microarrays (TMA). The Kaplan-Meier survival curves were similar to those obtained from protein analysis: cases with low expression of *GASC1* mRNA had 2.3 years shorter breast cancer specific survival than cases with high *GASC1* mRNA expression (p=0.132, Log Rank; Figure 6). *GASC1* mRNA expression in GASC1 negative cases was significantly lower than in GASC1 positive ones (Mann–Whitney: p=0.004). The expression of *GASC1* mRNA was significantly lower in grade II and III tumors compared with its expression in grade I tumors (Figure 7). This supports the previous finding from TMA analysis showing that the great majority (84%) of tumors of grade II and III were GASC1 negative.

**Figure 8:**
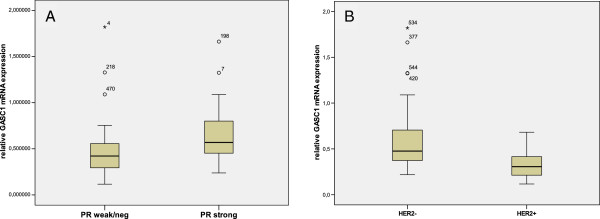
**Graphs showing *GASC1 *mRNA expression according to progesterone receptor (PR) and HER2 status. **(**A**) *GASC1 *mRNA relative level is lower in tumors showing negative or weak expression of PR (0.483±0.051) compared with tumors showing high PR expression (0.691±0.092; Mann–Whitney: p=0.016). (**B**) *GASC1* mRNA relative level is higher in HER2 negative tumors (0.599±0.055) than in HER2 positive tumors (0.326±0.054; Mann–Whitney: p=0.004).

The cases with negative or weak PR expression had a significantly lower relative level of *GASC1* mRNA than the cases with high PR expression (Mann–Whitney: p=0.016). In contrast, HER2 negative cases showed significantly higher *GASC1* mRNA expression than the HER2 positive counterparts (Mann–Whitney: p=0.004), which was in line with the protein staining results (Figure 8).

## Discussion

This is the first study investigating GASC1 status in a relatively large group of clinical samples (altogether 355 cases of invasive breast cancer). We show that GASC1 negativity is an independent prognostic factor of poorer breast cancer specific survival, not only overall but also in groups of patients treated with different adjuvant therapies, evidence of its prognostic as well as predictive value for women with invasive breast cancer.

GASC1 negative women had poorer breast cancer specific survival, when they were in more advanced stages of the disease (T2 – T4, positive lymph nodes, clinical stages II - IV). However, the survival of women in initial stages of the disease did not differ between GASC1 positive and negative cases, even though the percentage of GASC1 positive tumors in these women was similar to the percentage in women in more advanced stages of the disease (results not shown). This observation points to a modulating role for GASC1 in the regulation of gene expression rather than a clear-cut activating or inhibitory role. An interesting finding from the survival analysis was that HER2 negative patients survived significantly better when they had GASC1 positive tumors regardless of their clinical stage. This observation might point the search for new therapeutic possibilities in the direction towards the GASC1 signaling pathway. Identification of molecules involved in this pathway and elucidating their role in the breast cancer pathophysiology could well be beneficial especially for those patients with triple negative tumors.

Moreover, we observed that GASC1 negative cases overall and in advanced clinical stages had a shorter time to relapse than the GASC1 positive patients, which was in agreement with the breast cancer specific survival analysis. Further, we demonstrated that the GASC1 positivity was statistically significantly associated with improved relapse-free and breast cancer specific survival in patients who were treated with adjuvant radiotherapy or with adjuvant tamoxifen which supported the above results. Those patients with ER-positive, HER2-negative disease are the group in whom decisions about adjuvant chemotherapy are most difficult. The relative indications for chemoendocrine therapy and endocrine therapy alone are given by the St Gallen International Expert Consensus [1]. However, there are no guidelines for those ER-positive, HER2-negative patients who are node positive with grade II tumors of size 2.1 – 5 cm (T2-T3). Our results indicate that the patients with HER2 negative, grade II tumors of ductal type have better prognosis when they are GASC1 positive (Table 5). Moreover GASC1 positive patients respond better to hormonal treatment. The results suggest that GASC1 positivity in these patients might be an indication for endocrine therapy alone, however this will need to be confirmed in a larger group of patients.

The majority of GASC1 negative tumors were ductal cases of higher histological grade, more often ER and PR negative and HER2 positive. We confirmed these results by analysis of *GASC1* mRNA expression and we also excluded the possibility that GASC1 is only a marker for aggressive tumor subtype. As far as we are aware, there are no studies of IHC GASC1 expression in human breast cancer and therefore, we analyzed the list of genes which were affected by *GASC1* depletion in study of Loh at al. [17] (Supplementary Table 1 by Loh at al. [17]). Several genes which were up-regulated by *GASC1* depletion in this study have also been found to be up-regulated in certain human tumors. Gene Sp5 (fold change 4.8) was reported to be overexpressed in several human tumors including hepatocellular carcinoma, gastric cancer, and colon cancer [25,26]. Annexin A1 (*ANXA1*, fold change 7.1) has been shown to have oncogenic potential in breast cancer progression and metastasis [27] and was significantly correlated with unfavorable prognostic features of breast cancer [28]. *Insulin-like growth factor-2* (*IGF-2*, fold change 4.5) and *H19* (fold change 4.2) are located together within an imprinted domain of chromosome 11p15.5. Loss of imprinting of *IGF2* resulting in increased expression of IGF-2 is a common genetic alteration in human malignancies and aberrant methylation of *IGF2/H19* locus has been detected in multiple human cancers including breast cancer [29-31]. Overexpression of IGF-2 was also positively correlated with increased risk of breast cancer, nodal positivity and higher tumor grade [32]. *Forkhead box Q1* (*Foxq1*, fold change 6.7) gene expression was correlated with high-grade basal-like breast cancers where it was associated with metastases and poor clinical outcomes [33,34].

The above evidence suggests that the absence of GASC1 might cause over-expression of several genes responsible for carcinogenesis and a more aggressive tumor phenotype in different cancers including breast cancer, which supports our finding that GASC1 negativity is correlated with more aggressive breast tumors and poorer prognosis. Based on these results, we speculate that GASC1 and possibly its up- and/or downstream factors might help to define breast tumors with better or poorer prognosis, especially in those which are considered to be more aggressive. However this hypothesis needs confirmation in a larger group of patients.

GASC1 possesses enzymatic activity i.e. it specifically demethylates tri- and dimethylated (me3 and me2) lysine (K) in residues 9 and 36 on histone H3 (H3K9me3/2 and H3K36me3/2) [9,16,35] and together with histone methyltransferases, it dynamically modulates the methylation status of H3. The biological significance of H3 methylation depends on the modified residue (K9 or K36), the degree of methylation (me1, me2, me3) and the genomic position of H3 (promoter region or coding region) [36,37]. In general, H3K9me3/2 is found in promoter regions of inactive genes and demethylation in this site triggers promoter activation, whereas H3K36me3/2 is enriched within the body of active genes and demethylation in this site is related to the termination of transcription [10,38]. However, the increase of H3K9me3 inactive mark in coding region of the gene has also been associated with active gene expression [39].

The transcriptional repressive effect of the H3K9 methylation can be attributed to the association with the repressive protein HP1 (heterochromatin protein 1) [11]. Cloos at al. [9] reported that GASC1 could effectively demethylate H3K9me3 and H3K9me2, release the repressive protein HP1 and reduce heterochromatin in vivo. One could speculate that in the absence of GASC1 HP1 would be recruited to H3K9me3/2 and stabilize heterochromatin. Recent data have shown that HP1α is over-expressed in numerous cancers and that HP1α over-expression is associated with increased cell proliferation, most likely through silencing of genes inhibiting cell proliferation. Moreover, the same authors demonstrated that HP1α overexpression in breast cancer patient samples correlated with an increased risk of death [40]**.** These findings could explain, at least to some extent, why GASC1 negativity is associated with more aggressive tumors and poorer breast cancer specific survival.

H3K9me3 status is also mediated by different histone methyltransferases. Suv39H1 (suppressor of variegation 3–9 homolog 1) is a major methyltransferase responsible for H3K9me3 that is intimately linked to DNA methylation. Dong at al. (2012) have found that H3K9me3 and DNA methylation on the E-cadherin promoter were higher in basal-like breast cancer cell lines. Furthermore, they showed that knockdown of Suv39H1 restored E-cadherin expression by blocking H3K9me3 and DNA methylation and resulted in an inhibition of cell migration, invasion and metastasis of basal-like breast cancer [41]. GASC1 activity eliminates H3K9me3, which in view of the above data, might partly explain the protective role of GASC1 evident in our data.

The role of GASC1 in prostate cancer has been studied by Wissmann at al. [16]. GASC1 colocalizes with androgen receptor (AR) in both normal prostate and prostate carcinomas. It enhances the transcription of AR responsive genes, and this property may contribute to tumor cell proliferation. In primary breast carcinomas, including triple-negative breast cancer, AR expression was interestingly associated with a significantly better disease free interval and overall survival [42-44]. Moreover, AR positive breast tumors have been shown to be smaller, more often node negative, lower in histological grade and clinical stage than their AR negative counterparts [42-45]. The clinico-pathological characteristics of AR positive tumors in the above studies resemble the characteristics of the GASC1 positive tumors in our study. This suggests that the GASC1 and the AR might be functionally connected also in breast cancers. However, the effect of AR stimulation in breast tissue is reported to be opposite to AR stimulation in prostate [46].

Regardless of repressing or activating AR dependent genes, GASC1 as an epigenetic factor can have a more general influence on gene expression and it also may modulate the expression of genes which are not dependent on the AR.

GASC1 demethylase activity has been shown to regulate the expression of genes critical for stem cell self-renewal, including NOTCH1 and NANOG, and possibly to be linked to the stem cell phenotypes in breast cancer [13,17,18]. The mammary gland is a subject to many tissue remodeling events occurring during puberty, pregnancy, lactation and menopause. Therefore, there needs to be a compartment of normal stem cells or early progenitor cells with high proliferative potential and differentiation abilities in order to maintain the mammary gland function. It has been proposed that breast tumors arise from normal stem cells or early progenitor cells through deregulation of normal self-renewal [47]. It is possible that GASC1 maintains the normal population of stem cells through mechanisms connected with HP1 regulation as described above. This function of GASC1 might be responsible for a lower recurrence rate in GASC1 positive cases compared with the GASC1 negative and the better outcome of GASC1 positive patients treated with radiotherapy.

The results obtained from clinical material are not consistent with *in vitro* results from breast cancer cell lines. Liu at al. [13] reported that *GASC1* expression was significantly higher in those cell lines representing aggressive basal-like breast cancers compared with the cell lines representing nonbasal-like breast cancers. Moreover, they demonstrated that *GASC1* was able to induce transformed phenotypes when overexpressed in immortalized, non-transformed ER negative mammary epithelial MCF10A cells. In our clinical material, GASC1 positivity was associated with less aggressive tumors. The reason for this discrepancy might be that cell lines only generally reflect the pathophysiological environment of the tumor without including all the up- and downstream factors present in the organism that influence the tumor behavior. Liu and coworkers [13] linked the above oncogenic properties of *GASC1* with induction of NOTCH1 by GASC1. However, there is evidence that NOTCH proteins can act either as oncogenes or as tumor suppressors depending on the cellular context [48,49]. One reason for the difference between our results and the report of Liu at al. [13] is that the latter did not take into account the cellular context of GASC1 action i.e. stromal or physiological interactions were absent in the cell cultures.

In addition to cell culture experiments, Liu at al. [13] meta-analyzed data sets concerning gene expression in human breast cancer from ONCOMINE. The results concerning GASC1 mRNA expression from this meta-analysis were consistent with their results obtained from cell culture, but did not support our results from IHC analysis of GASC1 expression. The discrepancy might be attributed to differences in the studied material. The data set from Fiank at al. [50] concerned gene expression in tumor stroma, while we assessed GASC1 expression in the epithelial compartment of the tumor. The analysis which was based on material used by Kreike at al. [51] consisted of 58% triple negative/basal like carcinomas, while in our material, triple negative tumors constituted only 11% of the total[51]. The discrepancy might also come from differences in methodology. We performed immunohistochemistry and compared numbers of GASC1 positive and negative tumors in different histopathological and clinical groups, while Liu at al. based their work on *GASC1* mRNA and protein expression analysis in different experimental setups. Our sample size available for GASC1 mRNA expression study was too small to permit a detailed statistical analysis, which prohibits a relevant comparison between our *GASC1* expression results with the *GASC1* expression evaluated by others.

## Conclusions

In summary, this study shows that the histone demethylase, GASC1, can have clinical significance as a prognostic as well as a predictive factor, indicating better prognosis and better response to radiotherapy and adjuvant treatment. GASC1 can possess either tumor suppressing or oncogenic properties, which may be context and organ specific.

## Abbreviations

*ANXA1*: Annexin A1; AR: Androgen receptor; ABC: Avidin-biotin complex; DAB: Diaminobenzidine tetrahydrochloride; ER: Estrogen receptor; *Foxq1*: *Forkhead box Q1*; *GASC1*: Gene amplified in squamous cell carcinoma 1; GASC1: Histone demethylase for trimethylated lysine 9 and 36 on histone H3; GR: Grade; HER2: Human epidermal growth factor receptor 2; HP1: Heterochromatin protein 1; HR: Hazard ratio; H3K9me3/2/1: Tri-, di-, monomethylated lysine in residue 9 on histone H3; H3K36me3/2/1: Tri-, di-, monomethylated lysine in residue 36 on histone H3; IHC: Immunohistochemistry; *IGF-2*: Insulin-like growth factor-2; *PPIA*: Peptidylprolyl isomerase A; PR: Progesterone receptor; RT-PCR: Reverse transcription - polymerase chain reaction; SOX2: (Sex determining region Y)-box 2; Suv39H1: Suppressor of variegation 3–9 homolog 1; TMA: Tissue microarray.

## Competing interests

The authors declare that they have no competing interests.

## Authors’ contributions

BB evaluated immunostaining results, performed the statistical analyses, drafted the manuscript and was aided in this effort by YS and AM; KN isolated RNA and carried out the quantitative real time RT-PCR, processed and analyzed raw data of PCR; YS evaluated immunostaining results, performed the statistical analyses, supervised the immunohistochemical staining and critically revised the manuscript; MT participated in the data collection and interpretation and statistical analysis and wrote the clinical part of the manuscript; VMK participated in data collection and interpretation and critically revised the manuscript; MM and JJP critically revised the manuscript; AM participated in data analysis and interpretation, read the draft, and critically revised the manuscript; YS, VMK, JJP and AM designed the study. All authors read and approved the final manuscript.

## Pre-publication history

The pre-publication history for this paper can be accessed here:

http://www.biomedcentral.com/1471-2407/12/516/prepub

## References

[B1] Goldhirsch A Ingle JN Gelber RD Coates AS Thurlimann B Senn HJ Thresholds for therapies: highlights of the St Gallen International Expert Consensus on the primary therapy of early breast cancer 2009 Ann Oncol 2009 20 8 1319 1329 10.1093/annonc/mdp322 19535820 PMC2720818

[B2] Rodriguez-Paredes M Esteller M Cancer epigenetics reaches mainstream oncology Nat Med 2011 17 3 330 339 21386836 10.1038/nm.2305

[B3] Sharma S Kelly TK Jones PA Epigenetics in cancer Carcinogenesis 2010 31 1 27 36 10.1093/carcin/bgp220 19752007 PMC2802667

[B4] Stefanska B Karlic H Varga F Fabianowska-Majewska K Haslberger A Epigenetic mechanisms in anti-cancer actions of bioactive food components - the implications in cancer prevention Br J Pharmacol 2012 167 2 279 297 10.1111/j.1476-5381.2012.02002.x 22536923 PMC3481038

[B5] Van Neste L Herman JG Otto G Bigley JW Epstein JI Van Criekinge W The epigenetic promise for prostate cancer diagnosis Prostate 2012 72 11 1248 1261 10.1002/pros.22459 22161815

[B6] Chiam K Centenera MM Butler LM Tilley WD Bianco-Miotto T GSTP1 DNA methylation and expression status is indicative of 5-aza-2’-deoxycytidine efficacy in human prostate cancer cells PLoS One 2011 6 9 e25634 10.1371/journal.pone.0025634 21980513 PMC3182253

[B7] Gomori E Pal J Kovacs B Doczi T Concurrent hypermethylation of DNMT1, MGMT and EGFR genes in progression of gliomas Diagn Pathol 2012 7 8 10.1186/1746-1596-7-8 22264301 PMC3292961

[B8] Portela A Esteller M Epigenetic modifications and human disease Nat Biotechnol 2010 28 10 1057 1068 10.1038/nbt.1685 20944598

[B9] Cloos PA Christensen J Agger K Maiolica A Rappsilber J Antal T Hansen KH Helin K The putative oncogene GASC1 demethylates tri- and dimethylated lysine 9 on histone H3 Nature 2006 442 7100 307 311 10.1038/nature04837 16732293

[B10] Bannister AJ Schneider R Myers FA Thorne AW Crane-Robinson C Kouzarides T Spatial distribution of di- and tri-methyl lysine 36 of histone H3 at active genes J Biol Chem 2005 280 18 17732 17736 15760899 10.1074/jbc.M500796200

[B11] Bannister AJ Zegerman P Partridge JF Miska EA Thomas JO Allshire RC Kouzarides T Selective recognition of methylated lysine 9 on histone H3 by the HP1 chromo domain Nature 2001 410 6824 120 124 10.1038/35065138 11242054

[B12] Yang ZQ Imoto I Fukuda Y Pimkhaokham A Shimada Y Imamura M Sugano S Nakamura Y Inazawa J Identification of a novel gene, GASC1, within an amplicon at 9p23-24 frequently detected in esophageal cancer cell lines Cancer Res 2000 60 17 4735 4739 10987278

[B13] Liu G Bollig-Fischer A Kreike B van de Vijver MJ Abrams J Ethier SP Yang ZQ Genomic amplification and oncogenic properties of the GASC1 histone demethylase gene in breast cancer Oncogene 2009 28 50 4491 4500 10.1038/onc.2009.297 19784073 PMC2795798

[B14] Rui L Emre NC Kruhlak MJ Chung HJ Steidl C Slack G Wright GW Lenz G Ngo VN Shaffer AL Cooperative epigenetic modulation by cancer amplicon genes Cancer Cell 2010 18 6 590 605 10.1016/j.ccr.2010.11.013 21156283 PMC3049192

[B15] Wu J Liu S Liu G Dombkowski A Abrams J Martin-Trevino R Wicha MS Ethier SP Yang ZQ Identification and functional analysis of 9p24 amplified genes in human breast cancer Oncogene 2012 31 3 333 341 10.1038/onc.2011.227 21666724 PMC3886828

[B16] Wissmann M Yin N Muller JM Greschik H Fodor BD Jenuwein T Vogler C Schneider R Gunther T Buettner R Cooperative demethylation by JMJD2C and LSD1 promotes androgen receptor-dependent gene expression Nat Cell Biol 2007 9 3 347 353 10.1038/ncb1546 17277772

[B17] Loh YH Zhang W Chen X George J Ng HH Jmjd1a and Jmjd2c histone H3 Lys 9 demethylases regulate self-renewal in embryonic stem cells Genes Dev 2007 21 20 2545 2557 10.1101/gad.1588207 17938240 PMC2000320

[B18] Katoh Y Katoh M Comparative integromics on JMJD2A, JMJD2B and JMJD2C: preferential expression of JMJD2C in undifferentiated ES cells Int J Mol Med 2007 20 2 269 273 17611647

[B19] Kauppinen JM Kosma VM Soini Y Sironen R Nissinen M Nykopp TK Karja V Eskelinen M Kataja V Mannermaa A ST14 gene variant and decreased matriptase protein expression predict poor breast cancer survival Cancer Epidemiol Biomarkers Prev 2010 19 9 2133 2142 10.1158/1055-9965.EPI-10-0418 20716618

[B20] Pellikainen MJ Pekola TT Ropponen KM Kataja VV Kellokoski JK Eskelinen MJ Kosma VM p21WAF1 expression in invasive breast cancer and its association with p53, AP-2, cell proliferation, and prognosis J Clin Pathol 2003 56 3 214 220 10.1136/jcp.56.3.214 12610102 PMC1769912

[B21] Hartikainen JM Tuhkanen H Kataja V Dunning AM Antoniou A Smith P Arffman A Pirskanen M Easton DF Eskelinen M An autosome-wide scan for linkage disequilibrium-based association in sporadic breast cancer cases in eastern Finland: three candidate regions found Cancer Epidemiol Biomarkers Prev 2005 14 1 75 80 15668479

[B22] Soini Y Tuhkanen H Sironen R Virtanen I Kataja V Auvinen P Mannermaa A Kosma VM Transcription factors zeb1, twist and snai1 in breast carcinoma BMC Cancer 2011 11 73 10.1186/1471-2407-11-73 21324165 PMC3055233

[B23] Cancer TIAfRo Tavassoéli FA, Devilee P Pathology and Genetics of Tumours of the Breast and Female Genital Organs IARC WHO Classification of Tumours 2003 Lyon, France: IARCPress-WHO 432

[B24] McNeill RE Miller N Kerin MJ Evaluation and validation of candidate endogenous control genes for real-time quantitative PCR studies of breast cancer BMC Mol Biol 2007 8 107 10.1186/1471-2199-8-107 18042273 PMC2211316

[B25] Chen Y Guo Y Ge X Itoh H Watanabe A Fujiwara T Kodama T Aburatani H Elevated expression and potential roles of human Sp5, a member of Sp transcription factor family, in human cancers Biochem Biophys Res Commun 2006 340 3 758 766 10.1016/j.bbrc.2005.12.068 16380080

[B26] Takahashi M Nakamura Y Obama K Furukawa Y Identification of SP5 as a downstream gene of the beta-catenin/Tcf pathway and its enhanced expression in human colon cancer Int J Oncol 2005 27 6 1483 1487 16273202

[B27] Khau T Langenbach SY Schuliga M Harris T Johnstone CN Anderson RL Stewart AG Annexin-1 signals mitogen-stimulated breast tumor cell proliferation by activation of the formyl peptide receptors (FPRs) 1 and 2 FASEB J 2011 25 2 483 496 10.1096/fj.09-154096 20930115

[B28] Yom CK Han W Kim SW Kim HS Shin HC Chang JN Koo M Noh DY Moon BI Clinical significance of annexin A1 expression in breast cancer J Breast Cancer 2011 14 4 262 268 10.4048/jbc.2011.14.4.262 22323911 PMC3268921

[B29] Gebeshuber CA Martinez J miR-100 suppresses IGF2 and inhibits breast tumorigenesis by interfering with proliferation and survival signaling Oncogene 2012 10.1038/onc.2012.372 22926517

[B30] Yang F Bi J Xue X Zheng L Zhi K Hua J Fang G Up-regulated long non-coding RNA H19 contributes to proliferation of gastric cancer cells FEBS J 2012 279 17 3159 3165 10.1111/j.1742-4658.2012.08694.x 22776265

[B31] Amit D Hochberg A Development of targeted therapy for a broad spectrum of cancers (pancreatic cancer, ovarian cancer, glioblastoma and HCC) mediated by a double promoter plasmid expressing diphtheria toxin under the control of H19 and IGF2-P4 regulatory sequences Int J Clin Exp Med 2012 5 4 296 305 22993648 PMC3443897

[B32] Qiu J Yang R Rao Y Du Y Kalembo FW Risk factors for breast cancer and expression of insulin-like growth factor-2 (IGF-2) in women with breast cancer in Wuhan City China. PLoS One 2012 7 5 e36497 10.1371/journal.pone.0036497 PMC3360739 22662119

[B33] Qiao Y Jiang X Lee ST Karuturi RK Hooi SC Yu Q FOXQ1 regulates epithelial-mesenchymal transition in human cancers Cancer Res 2011 71 8 3076 3086 10.1158/0008-5472.CAN-10-2787 21346143

[B34] Zhang H Meng F Liu G Zhang B Zhu J Wu F Ethier SP Miller F Wu G Forkhead transcription factor foxq1 promotes epithelial-mesenchymal transition and breast cancer metastasis Cancer Res 2011 71 4 1292 1301 10.1158/0008-5472.CAN-10-2825 21285253 PMC3906209

[B35] Whetstine JR Nottke A Lan F Huarte M Smolikov S Chen Z Spooner E Li E Zhang G Colaiacovo M Reversal of histone lysine trimethylation by the JMJD2 family of histone demethylases Cell 2006 125 3 467 481 10.1016/j.cell.2006.03.028 16603238

[B36] Hoffmann I Roatsch M Schmitt ML Carlino L Pippel M Sippl W Jung M The role of histone demethylases in cancer therapy Mol Oncol 2012 10.1016/j.molonc.2012.07.004 PMC5528348 22902149

[B37] Shi Y Whetstine JR Dynamic regulation of histone lysine methylation by demethylases Mol Cell 2007 25 1 1 14 10.1016/j.molcel.2006.12.010 17218267

[B38] Barski A Cuddapah S Cui K Roh TY Schones DE Wang Z Wei G Chepelev I Zhao K High-resolution profiling of histone methylations in the human genome Cell 2007 129 4 823 837 10.1016/j.cell.2007.05.009 17512414

[B39] Peters AH Kubicek S Mechtler K O’Sullivan RJ Derijck AA Perez-Burgos L Kohlmaier A Opravil S Tachibana M Shinkai Y Partitioning and plasticity of repressive histone methylation states in mammalian chromatin Mol Cell 2003 12 6 1577 1589 10.1016/S1097-2765(03)00477-5 14690609

[B40] De Koning L Savignoni A Boumendil C Rehman H Asselain B Sastre-Garau X Almouzni G Heterochromatin protein 1alpha: a hallmark of cell proliferation relevant to clinical oncology EMBO Mol Med 2009 1 3 178 191 10.1002/emmm.200900022 20049717 PMC3378125

[B41] Dong C Wu Y Wang Y Wang C Kang T Rychahou PG Chi YI Evers BM Zhou BP Interaction with Suv39H1 is critical for Snail-mediated E-cadherin repression in breast cancer Oncogene 2012 10.1038/onc.2012.169 PMC3703513 22562246

[B42] Tang D Xu S Zhang Q Zhao W The expression and clinical significance of the androgen receptor and E-cadherin in triple-negative breast cancer Med Oncol 2012 29 2 526 33 10.1007/s12032-011-9948-2 21519872

[B43] Collins LC Cole KS Marotti JD Hu R Schnitt SJ Tamimi RM Androgen receptor expression in breast cancer in relation to molecular phenotype: results from the Nurses’ Health Study Mod Pathol 2011 24 7 924 931 10.1038/modpathol.2011.54 21552212 PMC3128675

[B44] Hu R Dawood S Holmes MD Collins LC Schnitt SJ Cole K Marotti JD Hankinson SE Colditz GA Tamimi RM Androgen receptor expression and breast cancer survival in postmenopausal women Clin Cancer Res 2011 17 7 1867 1874 10.1158/1078-0432.CCR-10-2021 21325075 PMC3076683

[B45] Niemeier LA Dabbs DJ Beriwal S Striebel JM Bhargava R Androgen receptor in breast cancer: expression in estrogen receptor-positive tumors and in estrogen receptor-negative tumors with apocrine differentiation Mod Pathol 2010 23 2 205 212 10.1038/modpathol.2009.159 19898421

[B46] Wang Y Romigh T He X Tan MH Orloff MS Silverman RH Heston WD Eng C Differential regulation of PTEN expression by androgen receptor in prostate and breast cancers Oncogene 2011 30 42 4327 4338 10.1038/onc.2011.144 21532617

[B47] Dontu G Al-Hajj M Abdallah WM Clarke MF Wicha MS Stem cells in normal breast development and breast cancer Cell Prolif 2003 36 Suppl 1 59 72 14521516 10.1046/j.1365-2184.36.s.1.6.x PMC6495427

[B48] Lobry C Oh P Aifantis I Oncogenic and tumor suppressor functions of Notch in cancer: it’s NOTCH what you think J Exp Med 2011 208 10 1931 1935 10.1084/jem.20111855 21948802 PMC3182047

[B49] Dumont AG Yang Y Reynoso D Katz D Trent JC Hughes DP Anti-tumor effects of the Notch pathway in gastrointestinal stromal tumors Carcinogenesis 2012 33 9 1674 1683 10.1093/carcin/bgs221 22764137 PMC3514902

[B50] Finak G Bertos N Pepin F Sadekova S Souleimanova M Zhao H Chen H Omeroglu G Meterissian S Omeroglu A Stromal gene expression predicts clinical outcome in breast cancer Nat Med 2008 14 5 518 527 10.1038/nm1764 18438415

[B51] Kreike B van Kouwenhove M Horlings H Weigelt B Peterse H Bartelink H van de Vijver MJ Gene expression profiling and histopathological characterization of triple-negative/basal-like breast carcinomas Breast Cancer Res 2007 9 5 R65 10.1186/bcr1771 17910759 PMC2242660

